# Effect of Fe-Doping on Thermal Expansion and Stability of Bismuth Magnesium Tantalate Pyrochlorere

**DOI:** 10.3390/ma15217668

**Published:** 2022-10-31

**Authors:** Nadezhda A. Zhuk, Maria G. Krzhizhanovskaya, Sergey V. Nekipelov, Viktor N. Sivkov, Danil V. Sivkov

**Affiliations:** 1 Institute of Natural Sciences, Syktyvkar State University, Oktyabrsky Prospect, 55, 167001 Syktyvkar, Russia; 2 Institute of Earth Sciences, Saint Petersburg State University, University Emb. 7/9, 199034 St. Petersburg, Russia; 3 Institute of Physics and Mathematics of the Komi Science Center UB RAS, Oplesnina St. 4, 167982 Syktyvkar, Russia

**Keywords:** bismuth tantalate pyrochlore, iron, FeMg codoping, thermal expansion, thermal stability

## Abstract

A continuous series of solid solutions (Bi_1.5_Mg_0.75−x_Fe_x_Ta_1.5_O_7±Δ_ (x = 0–0.75)) with the pyrochlore structure were synthesized with the solid-phase method. It was shown that iron, like magnesium, is concentrated in the structure in the octahedral position of tantalum. Doping with iron atoms led to an increase in the upper limit of the thermal stability interval of magnesium-containing pyrochlore from 1050 °C (x = 0) up to a temperature of 1140 °C (x = 1). The unit cell constant *a* and thermal expansion coefficient (TEC) increase uniformly slightly from 10.5018 Å up to 10.5761 Å and from 3.6 up to 9.3 × 10^−6^ °C^−1^ in the temperature range 30–1100 °C. The effect of iron(III) ions on the thermal stability and thermal expansion of solid solutions was revealed. It has been established that the thermal stability of iron-containing solid solutions correlates with the unit cell parameter, and the lower the parameter, the more stable the compound. The TEC value, on the contrary, is inversely proportional to the cell constant.

## 1. Introduction

Bismuth-containing compounds, in particular, pyrochlores, are promising due to their excellent dielectric properties—high dielectric permittivity, low dielectric losses in the MHz frequency range, and adjustable temperature coefficient of capacitance [1,2,3,4,5]. Pyrochlore-type A_2_M_2_O_7_ oxides have been proposed as excellent candidates for the immobilization of actinides [6]. At the same time, they are favorably distinguished by mild synthesis conditions and thermal stability in air over a wide temperature range, as well as chemical compatibility with low-melting and relatively cheap Cu, Ag conductors [7,8,9,10]. In this regard, materials based on such pyrochlores can be used in the manufacture of multilayer ceramic capacitors, dielectric resonators, thermistors, thick-film resistors and communication elements, microwave generators or filters [3,10,11,12]. In addition, recently there have been reports that pyrochlores can be used as thermal barrier materials and are promising for the manufacture of refractories, heat-insulating coatings, and fuel cells based on them. An example of this is lanthanum zirconate La_2_Zr_2_O_7_, which is featured with a low thermal conductivity, thermal and phase stability above 1400 °C [13]. Iron-containing pyrochlores based on bismuth niobate and tantalate are also characterized by thermal stability and the absence of phase transitions up to 1140 °C according to the thermal analysis of samples [14,15,16,17]. Meanwhile, the thermal behavior of iron- and magnesium-containing pyrochlores in the high-temperature range remains unstudied. In our article, we report on the production of a new continuous series of Bi_1.5_Mg_0.75−x_Fe_x_Ta_1.5_O_7±__Δ_ (x = 0–0.75) and the observed effect of iron ions on the thermal stability and thermal expansion of the bismuth magnesium tantalate pyrochlore. The thermal expansion of ceramics up to 1200 °C was studied and a comparative analysis of the data for Fe-doped and Fe,Mg-codoped bismuth tantalate pyrochlores was carried out. In particular, it has been found that an increase in the iron content in the samples leads to an increase in the upper limit of the stability temperature range from 1050 up to 1140 °C (x = 0.75). Pyrochlores Bi_1.5_Mg_0.75−x_Fe_x_Ta_1.5_O_7±Δ_ expand isotropically and weakly with increasing temperature. A symbatic behavior of the dependences of the pyrochlore cell constant on temperature and the degree of substitution of a small cation (Fe^3+^) for a larger one (Mg^2+^) was noted.

## 2. Experimental

Solid solutions of Bi_1.5_Mg_0.75−x_Fe_x_Ta_1.5_O_7±Δ_ (x = 0, 0.225, 0.375, 0.525, 0.75) were synthesized by the solid-phase method according to the procedure described in [18,19,20]. The microstructure and local elemental analysis were studied by scanning electron microscopy and energy-dispersive X-ray spectroscopy (electron scanning microscope Tescan VEGA 3LMN (Tescan, Czech Republic), energy dispersion spectrometer INCA Energy 450, Tescan, Czech Republic).

The thermal behavior of Fe,Mg-codoped bismuth tantalate at high temperature was investigated by the powder high-temperature X-ray diffraction (HTXRD) using a Rigaku Ultima IV diffractometer (Rigaku, Japan) (CoKα radiation (λ = 1.7902 Å), 40 kV/30 mA, Bragg-Brentano geometry, PSD D-Tex Ultra) with a thermo-attachment in the range of 30−1200 °C (air) with the T steps of 30 °C. Fine-powdered samples were deposited on a platinum sample holder (20 × 12 × 1.5 mm) from a heptane suspension. The temperature was controlled by a thermocouple located close to the Pt holder; the uncertainty of temperature measurement does not exceed 10 °C. The correctness of 2θ at room temperature was checked before every measurement using silicon as external standard; the change of zero-shift has never been more than ±0.02° 2θ in the whole temperature range. The unit-cell parameters were calculated at every temperature step by Pawley approach with the program package Topas 5.0 (Bruker AXS *GmbH*, Karlsruhe, Germany) [21]. The calculation of the thermal-expansion tensor was performed using the TTT [22] program package.

## 3. Results and Discussion

### 3.1. Morphology and Crystal Structure

**Figure 1:**
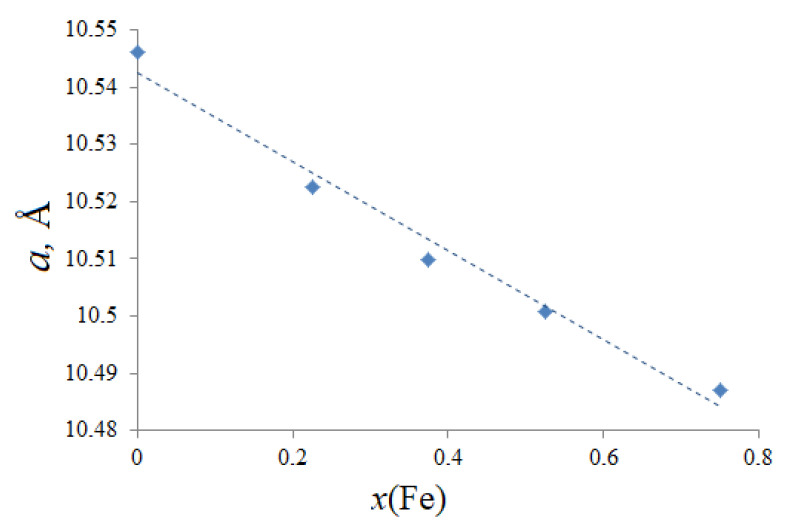
Dependence of the unit cell parameter on the content of iron ions in Bi_1.5_Mg_0.75−x_Fe_x_Ta_1.5_O_7±Δ_ solid solutions.

Samples of composition of Bi_1.5_Mg_0.75−x_Fe_x_Ta_1.5_O_7±Δ_ (x = 0, 0.225, 0.375, 0.525, 0.75), according to the results of X-ray phase analysis, crystallize in the cubic crystal system. An analysis of reflection extinctions made it possible to confirm that the symmetry of the crystal structure is indeed cubic with the space group $Fd\bar{3}m$:2. The structure of pyrochlore based on bismuth tantalate was refined by us earlier [17,18,19,20] and is used in this work to analyze the effect of iron atoms on the thermal expansion of samples. As a result of the calculation of the unit cell parameter of the samples, it was found that with an increase in the concentration of iron and a decrease in the content of magnesium, the cell constant decreases uniformly from 10.5225 ± 0.0007 (x = 0.225) down to 10.5009 ± 0.0001 Å (x = 0.525), obeying the Vegard law. This fact, firstly, indicates the formation of a continuous series of solid solutions and, secondly, the same type and uniform distribution of iron and magnesium ions in one system of crystallographic sites, for example, only in the tantalum(V) octahedral sites. The dependence of the unit cell parameter on the iron content is shown in Figure 1.

It should be noted that the unit cell parameters previously determined by us for the end members of the series of the investigated Bi_1.5_Mg_0.75−x_Fe_x_Ta_1.5_O_7±Δ_ solid solution are a = 10.4871(2) Å (x = 0.75) [20] and a = 10.54607 Å (x = 0) [9,23], which is in good agreement with the parameters of iron- and magnesium-containing solid solutions. The fulfillment of Vegard’s rule also indicates that, regardless of concentration, magnesium and iron ions occupy the same cationic positions; apparently, position B, as was established for a solid solution with x = 0.75 according to Mössbauer spectroscopy data [20]. The decrease in the unit cell parameter of Bi_1.5_Mg_0.75−x_Fe_x_Ta_1.5_O_7±Δ_ solid solutions can be explained by taking into account the distribution of atoms and their ionic radii. First of all, we note that we consider only Fe(III) ions, since it was proved in [14,20,24,25,26] by the methods of magnetic susceptibility, Mössbauer, and Electron paramagnetic resonance (EPR) spectroscopy that there are no Fe(II) ions in synthesized pyrochlores. In our previous work [19], we showed that magnesium atoms occupy the positions of tantalum, causing an increase in the unit cell parameter, since the radius of tantalum(V) is smaller than the radius of magnesium(II) ions (R(Mg(II))c.n-6 = 0.72 Å, R(Ta(V))c.n-6 = 0.64 Å) [27]. In its turn, the unit cell parameter of the solid solutions under consideration decreases with a decrease in the magnesium content, which may mean that magnesium ions are replaced by iron(III) ions in the crystallographic positions of tantalum(V) rather than bismuth(III) (R(Fe(III))c.n-6 = 0.645 Å, R(Bi(III))c.n-6 = 1.17 Å). It was previously established that the unit cell parameter of iron-containing solid solutions slightly increases with increasing iron content; for example, for Bi_3.36_Fe_2.08+x_Ta_2.56-x_O_14.56-x_ (−0.32 ≤ x ≤ 0.48) from 10.4979(8) up to 10.5033(1) Å, for Bi_3.36_Fe_2.08+x_Nb_2.56-x_O_14.56-x_ (−0.24 ≤ x ≤ 0.48) from 10.5071(4) up to 10.5107(7) Å, for Bi_3.36_Fe_2.08+x_Sb_2.56-x_O_14.56-x_ (0 ≤ x ≤ 0.64) from 10.4284(8) up to 10.4513(8) Å, emphasizing the possibility of replacing Nb/Ta/Sb ions with iron(III) ions [14,15,16,17,28]. In this case, the increase in the cell parameter is explained by the slightly larger ionic radius of iron(III) compared to the radii of niobium(V), tantalum(V), and antimony(V) (R(Fe(III))c.n-6 = 0.645 Å, R(Sb(V))c.n-6 = 0.60 Å, R(Nb(V))c.n-6 = 0.64 Å, R(Ta(V))c.n-6 = 0.64 Å) [27]. In general, the lattice constant for the solid solutions studied by us satisfactorily fits into the range of values for iron-containing pyrochlores based on bismuth tantalate.

**Figure 2:**
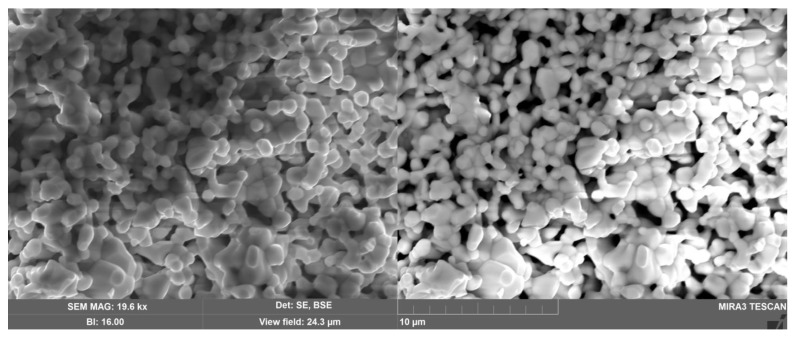
SEM micrographs of Bi_1.5_Mg_0.75−x_Fe_x_Ta_1.5_O_7−Δ_ (x = 0.375) ceramics.

The color of the samples is yellow, characteristic of iron(III) compounds. The microstructure of the samples is porous, it is a complex cellular structure formed by weakly aggregated elongated particles 0.5–3 µm in size (Figure 2). The dependence of the crystallite size on the iron content is not reliably detected. It can be precisely noted that doping with iron(III) ions does not significantly change the porous microstructure of magnesium pyrochlore. Local quantitative analysis by the EDS method showed that the experimental composition of the samples corresponded to the specified one (Figure S1).

**Figure 3:**
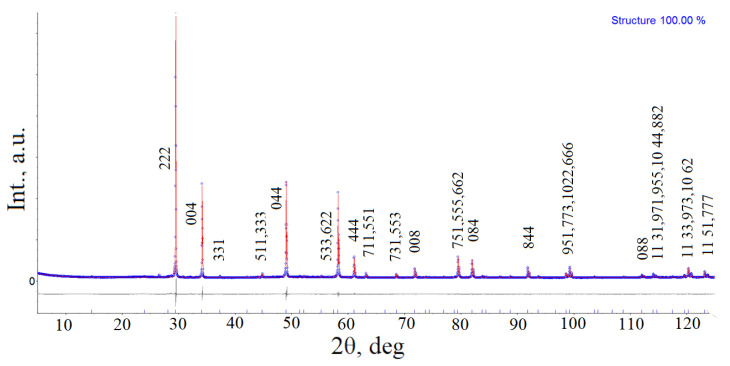
Experimental (blue circles), calculated (solid red line) and difference (grey line) XRD patterns of Bi_1.5_Mg_0.375_Fe_0.375_Ta_1.5_O_7−Δ_.

Based on powder XRD data, the crystal structure was refined for the composition Bi_1.5_Mg_0.375_Fe_0.375_Ta_1.5_O_7−Δ_ using the Topas 5.0 software package [21]. Table 1 and Table 2 show the results of the refinement of the structure of pyrochlore by the Rietveld method in the space group Fd3 m:2 (227). The experimental, calculated, and difference diffraction patterns of Bi_1.5_Mg_0.375_Fe_0.375_Ta_1.5_O_7−Δ_ are shown in Figure 3.

The cell parameter of pyrochlore calculated by us does not contradict the values given in articles [14,15,16,17] for iron-containing pyrochlores based on tantalate (10.4979–10.5033 Å) and (Bi_1.721_Fe_0.190_(Fe_0.866_Nb_1.134_)O_7_) a = 10.508 of bismuth niobate.

**Figure 4:**
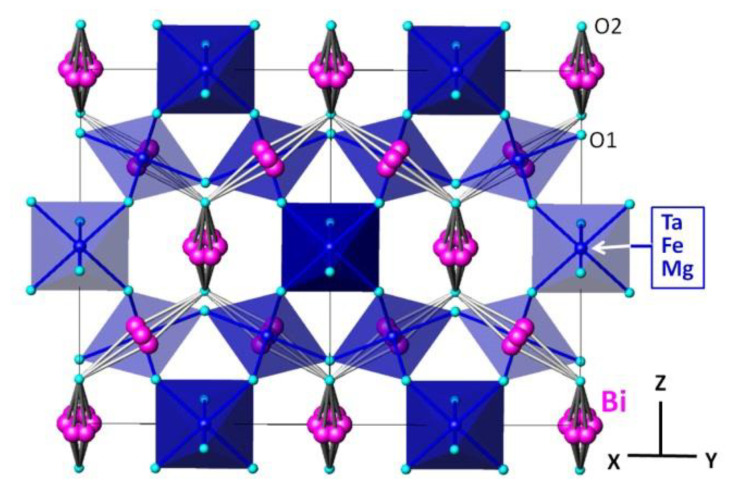
The projection of Bi_1.5_Mg_0.75−x_Fe_x_Ta_1.5_O_7±Δ_ (x = 0.375) pyrochlore crystal structure onto (110) crystallographic plane.

According to the simulation results, the tantalum/iron/magnesium atoms occupy positions 16b and form a regular octahedron MO_6_ (M = Fe, Mg, Ta) with a bond length M-O~1.996 Å (Table 3, Figure 4). The above assumption about the octahedral positions of Fe(III) ions does not contradict the results of Mössbauer spectra studies of iron-containing pyrochlores. In particular, for the structural analogue of Bi_2_FeTa_2_O_9_, the parameters of the Mössbaur spectrum of iron ions, which are characteristic of Fe(III) ions in octahedral coordination, were previously determined: IS = 0.365 ± 0.0020 mm/s, QS = 0.604 ± 0.034 mm/s [20]. Individual interatomic distances in the weakly BiO_8_ polyhedron vary from 2.31 up to 2.98 Å (Table 3), with 4 out of 8 bonds noticeably shorter than the others. The asymmetry of the bismuth ion polyhedron can be due to the presence of a stereoactive 6s^2^ lone pair. Around the incompletely occupied position of the O(2) atom, one can distinguish an anion-centered tetrahedron formed by bismuth atoms O(2)Bi_4_.

It should be noted that the geometric parameters of the Bi_1.5_Mg_0.375_Fe_0.375_Ta_1.5_O_7−Δ_ pyrochlore structure are slightly larger than those for Bi_1.5_Fe_0.75_Ta_1.5_O_7+Δ_ [17], which is due to the difference between the radii of Mg(II) and Ta(V), Fe(III). For example, the average Ta-O bond length for Bi_1.5_Fe_0.75_Ta_1.5_O_7+Δ_ is ~1.987 Å, while the Bi-O distances in the polyhedron vary from 2.30 up to 2.99 Å

### 3.2. Thermal Stability

**Figure 5:**
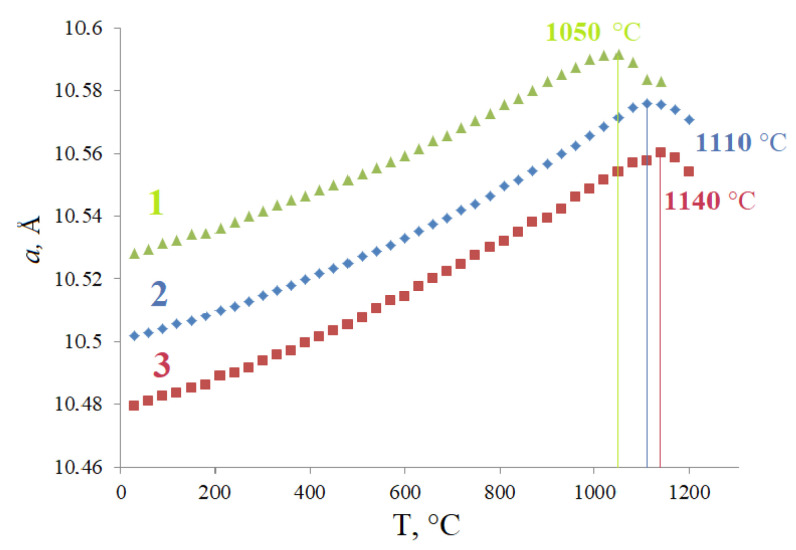
Temperature dependences of unit cell parameters of Bi_1.5_Mg_0.75_Ta_1.5_O_7_ (1), Bi_1.5_Mg_0.375_Fe_0.375_Ta_1.5_O_7−Δ_ (2)_,_ Bi_1.5_Fe_0.75_Ta_1.5_O_7+Δ_ (3) pyrochlores.

The study of thermal behavior and stability of solid solutions was carried out by the HTXRD method in the range of 30–1200 °C (Tables S1 and S2). Figure 5 shows the temperature dependence of the cubic unit cell parameter of a solid solution with an equal proportion of magnesium and iron (x = 0.375) for the range of 30–1200 °C in comparison with the cell parameter for the compounds that are the extreme members of the solid solution series: Bi_1.5_Mg_0.75_Ta_1.5_O_7−Δ_ and Bi_1.5_Fe_0.75_Ta_1.5_O_7+Δ_. The unit cell parameter *a* increases uniformly from 10.50183 Å (30 °C) up to 10.57607 Å (1110 °C) (Table S1). As can be seen from Figure 4, the following relation holds between the unit cell parameters: *a*(Bi_1.5_Mg_0.75_Ta_1.5_O_7−Δ_) > *a*(Bi_1.5_Mg_0.375_Fe_0.375_Ta_1.5_O_7−Δ_) > *a*(Bi_1.5_Fe_0.75_Ta_1.5_O_7+Δ_). This fact can be explained by the replacement of ions of larger radius Mg(II) in octahedral coordination by ions with smaller radius–iron ions in the oxidation state (+3) (R(Mg(II))c.n-6 = 0.72Å, R(Fe(III))c.n-6 = 0.645Å, R(Fe(II))c.n-6 = 0.78Å). The unit cell parameter of Bi_1.5_Mg_0.375_Fe_0.375_Ta_1.5_O_7−Δ_ changes continuously up to a temperature of 1110 °C. A uniform change in the cell constant indicates the absence of phase transformations and thermal stability in the considered temperature range (30–1100 °C). Above 1110 °C, the thermal dissociation of the solid solution probably occurs, which was previously noted for Bi_1.5_Fe_0.75_Ta_1.5_O_7+Δ._ It also follows from the figure that the temperature of the upper limit of the thermal stability interval of Bi_1.5_Mg_0.375_Fe_0.375_Ta_1.5_O_7−Δ_ is 60 °C higher than for Bi_1.5_Mg_0.75_Ta_1.5_O_7−Δ,_ and 30 °C lower compared to Bi_1.5_Fe_0.75_Ta_1.5_O_7+Δ_. Based on the observed fact, it can be stated that doping with iron(III) ions increases the thermal stability of magnesium-containing pyrochlore.

This interesting fact can be explained in terms of covalence and Fe-O bond length in the FeO_6_ polyhedron. The fact is that the Fe(III)-O bond in the octahedron is much stronger than Mg(II)-O, since it is more covalent and shorter (R(MgO) = 2.007 Å, R(FeO = 1.996 Å)) [9,17,23]. This is indicated by the smaller unit cell parameter of Bi_1.5_Fe_0.75_Ta_1.5_O_7+Δ_ in comparison with Bi_1.5_Mg_0.75_Ta_1.5_O_7−Δ_.

**Figure 6:**
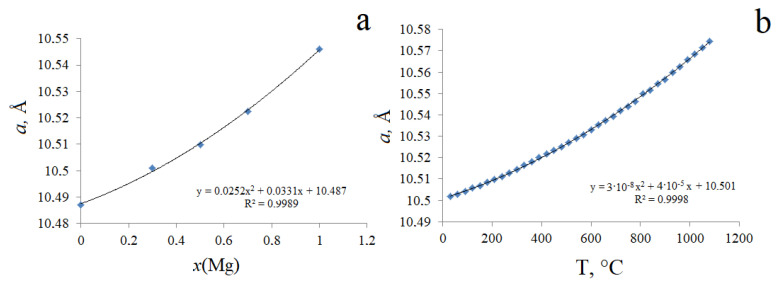
Similarity of the nature of the change in the lattice parameter of Bi_1.5_Fe_0.75_Ta_1.5_O_7+Δ_ upon substitution of Fe↔Mg (**a**) and under the influence of temperature (**b**).

In addition, if magnesium(II) ions are distributed in the Ta(V) positions, then the number of oxygen vacancies, and, consequently, distortions of the coordination polyhedron, will be more significant compared to Fe(III). These factors adversely affect the thermal stability of pyrochlore. As a result, it can be stated that the thermal stability of pyrochlore will be the higher, the smaller the parameter of its unit cell. One can also note a noticeable similarity in the nature of the change in the cell parameter when a smaller Fe(III) cation is replaced by a larger Mg(II) and with an increase in temperature (Figure 6). The figure shows that both dependencies are well described by a quadratic function with a high value of the correlation coefficient. The similarity of the effect of temperature and substitution of a smaller cation for a larger one is described for different classes of compounds. In particular, the similarity of thermal, baric, and compositional deformations was first noted in [29,30,31] and later by many authors, including [32,33,34]. The fact is that the substitution of cations of a smaller radius for larger ions is associated with an increase in length and, at the same time, a decrease in the strength of the bond, and an increase in stresses in the structure. This, in turn, has a destabilizing effect on the structure, similar to temperature effects (heating). Therefore, magnesium-containing pyrochlores, if heated, dissociate at a lower temperature than iron-containing pyrochlores.

As a result of the approximation of the temperature dependences of the unit cell parameters by a polynomial of the second degree, the values of the thermal expansion coefficients (TEC) α along the crystallographic direction were calculated at different temperatures (Table S2). Figure 6 shows the temperature dependences of the thermal expansion coefficient for Bi_1.5_Mg_0.375_Fe_0.375_Ta_1.5_O_7−Δ_ and for comparison Bi_1.5_Mg_0.75_Ta_1.5_O_7−Δ_, Bi_1.5_Fe_0.75_Ta_1.5_O_7+Δ_.

According to the results of the study of thermal expansion, pyrochlore Bi_1.5_Mg_0.375_Fe_0.375_Ta_1.5_O_7−Δ_ can be considered a weakly expanding compound with isotropic thermal expansion [17,18,19,23,35,36,37]. The isotropy of thermal expansion is due to the cubic symmetry of the crystal lattice. The value of TEC for Bi_1.5_Mg_0.375_Fe_0.375_Ta_1.5_O_7−Δ_ is increased uniformly from 3.6 up to 9.3 × 10^−6^ °C^−1^ in the temperature range of 30–1050 °C. The average TEC value in the range of 30–1050 °C is 6.4 × 10^−6^ °C^−1^. The average value of TEC is comparable with the thermal expansion coefficient for the majority of compounds with pyrochlore frame structure [35,36,37] and earlier studied by us pyrochlores of composition Bi_2_NiTa_2_O_9,_ Bi_2_MgTa_2_O_9_, Bi_2_FeTa_2_O_9+Δ_ [17,18,19,23], confirming the thesis about the weak effect of the nature of dopants distributed in the three-dimensional cationic sublattice B on the thermal expansion of framework crystalline structures of the pyrochlore type.

**Figure 7:**
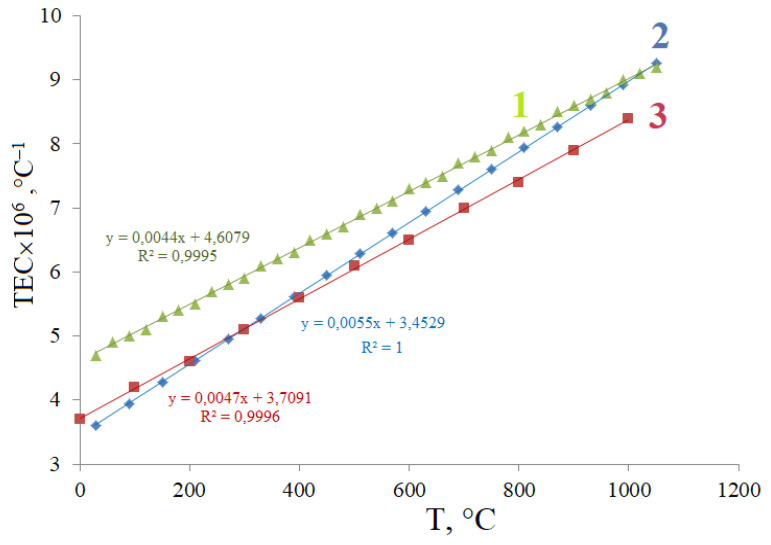
Temperature dependences of TECs of Bi_1.5_Mg_0.75_Ta_1.5_O_7_ (1), Bi_1.5_Mg_0.375_Fe_0.375_Ta_1.5_O_7−Δ_ (2) and Bi_1.5_Fe_0.75_Ta_1.5_O_7+Δ_ (3) pyrochlores.

It is interesting to note that if the change in the unit cell parameter of doped pyrochlores changes regularly and depends on the nature and length of the chemical bond formed by the dopant and oxygen atoms in the coordination polyhedron, then the prediction of the TEC values and the course of their temperature dependences is not so obvious. As can be seen from Figure 7, the Bi_1.5_Fe_0.75_Ta_1.5_O_7+Δ_, compound has the highest values of TEC in the entire temperature range, and Bi_1.5_Mg_0.75_Ta_1.5_O_7_ has the lowest values, intermediate values are taken by the TEC for Bi_1.5_Mg_0.375_Fe_0.375_Ta_1.5_O_7−Δ_. Apparently, the TEC values are inversely proportional to the unit cell parameter of pyrochlores and, by the way, to thermal stability, for comparison, *a* =10.4871 Å (Bi_1.5_Fe_0.75_Ta_1.5_O_7+Δ_), *a* = 10.5098 Å (Bi_1.5_Mg_0.375_Fe_0.375_Ta_1.5_O_7−Δ_) and *a* = 10.5282 Å (Bi_1.5_Mg_0.75_Ta_1.5_O_7_). This can be explained by the fact that a structure with a smaller unit cell parameter is characterized by smaller bond lengths between atoms. The rate of change in TEC with temperature for the compositions Bi_1.5_Fe_0.75_Ta_1.5_O_7+Δ_, Bi_1.5_Mg_0.75_Ta_1.5_O_7_ is approximately the same (the slope of the straight lines is 0.0044 and 0.0047, respectively). This may mean that these systems are characterized by the same type of thermal expansion of the three-dimensional octahedral framework. Their temperature dependences are almost parallel, which cannot be said about Bi_1.5_Mg_0.375_Fe_0.375_Ta_1.5_O_7−Δ_. The fact is that in the low-temperature region, Bi_1.5_Mg_0.375_Fe_0.375_Ta_1.5_O_7−Δ_ expands like Bi_1.5_Mg_0.75_Ta_1.5_O_7,_ and in the high-temperature region (near 1000 °C) its TEC (8.9 × 10^−6^ °C^−1^) is comparable with the TEC for Bi_1.5_Fe_0.75_Ta_1.5_O_7+Δ_ (9.0 × 10^−6^ °C^−1^). We do not consider this behavior to be an accident or a measurement error, since the compounds under consideration were synthesized and studied using a similar technique. It can be assumed that during thermal deformation of the solution, first of all, less covalent bonds of the Mg(II)-O type increase, as the least rigid ones, and then, with rise in temperature, more rigid more covalent bonds of the Fe(III)-O type are involved as well.

Thus, atoms of dopants (transition elements) cannot significantly affect the mechanism of thermal expansion of the framework structure of pyrochlore, but one can assume a selective effect of the nature of dopants on the nature of chemical bonds, thermal expansion, parameters, and stability of the crystal structure as a whole.

## 4. Conclusions

The thermal expansion and stability of iron-containing Bi_1.5_Mg_0.75−x_Fe_x_Ta_1.5_O_7±Δ_ solid solutions with the pyrochlore structure. A continuous series of solid solutions are formed, the unit cell parameter decreases with increasing iron(III) content. The effect of Fe(III) ions on the thermal stability of solid solutions has been established. An increase in the iron content in the samples leads to an increase in the upper limit of the stability temperature range from 1050 up to 1140 °C (x = 0.75). Pyrochlores Bi_1.5_Mg_0.75−x_Fe_x_Ta_1.5_O_7±Δ_ expand isotropically and weakly with an increasing temperature. A symbatic behavior of the dependences of the pyrochlore cell constant on temperature and the degree of substitution of a small cation (Fe^3+^) for a larger one (Mg^2+^) was noted. The average value of TEC in the range of 30–1050 °C (for x = 0.375) is 6.4 × 10^−6^ °C^−1^. For magnesium- or iron-containing pyrochlore, the TEC values in the studied temperature range are proportional to the iron content: For Bi_1.5_Fe_0.75_Ta_1.5_O_7+Δ_ they are greater than Bi_1.5_Mg_0.75_Ta_1.5_O_7_. Solid solutions of Bi_1.5_Mg_0.75−x_Fe_x_Ta_1.5_O_7±Δ_ in the low-temperature region expand similarly to Bi_1.5_Mg_0.75_Ta_1.5_O_7_, and in the high-temperature region similarly to Bi_1.5_Fe_0.75_Ta_1.5_O_7+Δ_. In the future, we plan to test the hypothesis of a significant effect of ions distributed in bismuth positions on the thermal expansion of pyrochlores.

## Figures and Tables

**Table 1:** Crystallographic data of Bi_1.5_Mg_0.375_Fe_0.375_Ta_1.5_O_7−Δ_.

*a* (Å)	10.51039(4)
α, β, γ (°)	90, 90, 90
*V* (Å^3^)	1161.064(12)
D_calc_ (g/cm^3^)	7.523(2)
*R*_B_ (%)	0.57
*Rwp* (%)	3.63
*Rp* (%)	2.63
*Rexp* (%)	2.16
*GOF*	1.68

**Table 2:** Parameters of iron-doped bismuth tantalate atoms.

Atom	Wyckoff Site	*x*	*y*	*z*	SOF	*B*_iso,Å_^2^
Bi	96g	0	−0.0252(1)	0.0252(1)	0.113(1)	1.00(7)
Ta	16b	0.5000	0.5000	0.5000	0.667(6)	0.62(3)
Fe	16b	0.5000	0.5000	0.5000	0.167(6)	0.62(3)
Mg	16b	0.5000	0.5000	0.5000	0.167(6)	0.62(3)
O1	48f	0.1250	0.1250	0.4306(5)	1	1.9(2)
O2	8a	0.1250	0.1250	0.1250	0.69(3)	1.9(2)

**Table 3:** Selected bond lengths in the structure of Bi_1.5_Mg_0.375_Fe_0.375_Ta_1.5_O_7__−__Δ_.

Bond	Length (Å)
Bi1–O1 × 2	2.3062(2)
–O1 × 2	2.344(4)
–O1 × 2	2.683(3)
–O2 × 2	2.983(4)
<Bi1_VIII_–O>	2.57905
Ta1–O1 × 6	1.9959(16)
<Ta1_VI_–O>	2.00

## Data Availability

Not applicable.

## Supplementary Materials

The following supporting information can be downloaded at: https://www.mdpi.com/article/10.3390/ma15217668/s1 Figure S1: EDS spectrum and microphotograph of the Bi_1.5_Mg_0.375_Fe_0.375_Ta_1.5_O_7−Δ_; Table S1: The unit cell parameters and volume values of Bi_1.5_Mg_0.375_Fe_0.375_Ta_1.5_O_7−Δ_ at different temperatures; Table S2: TECs of Bi_1.5_Mg_0.375_Fe_0.375_Ta_1.5_O_7−Δ_ at different temperatures.
