# Supplementary material for: Effect of Fe-Doping on Thermal Expansion and Stability of Bismuth Magnesium Tantalate Pyrochlorere
Source: Materials (Basel). 2022 Oct 31;15(21):7668. doi: 10.3390/ma15217668 (PMC9657759; doi:10.3390/ma15217668)
Supplement: Supplementary file 1 [file materials-15-07668-s001.zip › materials-1966842-supplementary.pdf]

Supplementary Information

Effect of Fe-Doping on Thermal Expansion and Stability of Bismuth Magnesium Tantalate Pyrochlorere

Nadezhda A. Zhuk [1,*], Maria G. Krzhizhanovskaya [2], Sergey V. Nekipelov [3], Viktor N. Sivkov [3] and Danil V. Sivkov [3]

[1] Institute of Natural Sciences, Syktyvkar State University, Oktyabrsky Prospect, 55, 167001 Syktyvkar, Russia

[2] Institute of Earth Sciences, Saint Petersburg State University, University Emb. 7/9, 199034 St. Petersburg, Russia; krzhizhanovskaya@mail.ru

[3] Institute of Physics and Mathematics of the Komi Science Center UB RAS, Oplesnina st. 4, 167982 Syktyvkar, Russia

* Correspondence: nzhuck@mail.ru

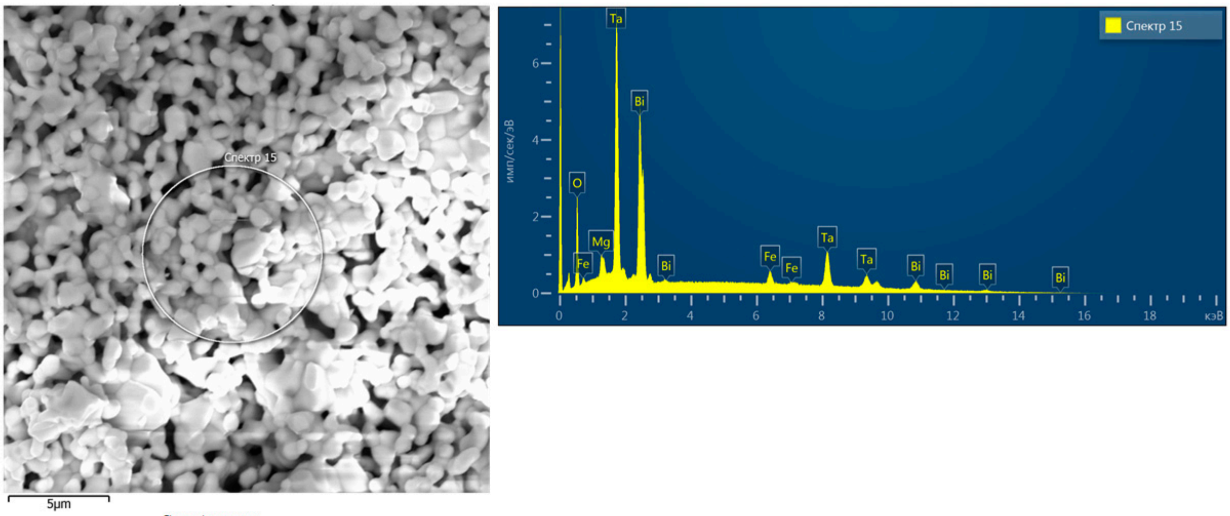

Spectrum 15

Element	Type of line	Weight %	Sigma Weight %	Atom. %
O	K Series	20.30	0.26	72.13
Ta	L Series	36.19	0.38	11.37
Fe	K Series	2.62	0.10	2.67
Bi	M Series	39.58	0.34	10.76
Mg	K Series	1.32	0.06	3.07
Total		100.00		100.00

Figure S1. EDS spectrum and microphotograph of the $Bi_{1.5}Mg_{0.375}Fe_{0.375}Ta_{1.5}O_{7-\Delta}$.

Table S1. The unit cell parameters and volume values of $Bi_{1.5}Mg_{0.375}Fe_{0.375}Ta_{1.5}O_{7-\Delta}$ at different temperatures.

T, ˚C	*a*, Å	*Δa*, Å	*V*, Å^3	*ΔV*, Å^3
30	10.50183	0.00015	1158.232	0.05
60	10.50278	0.00015	1158.546	0.05
90	10.50404	0.00010	1158.963	0.03
120	10.50570	0.00014	1159.511	0.05
150	10.50676	0.00015	1159.864	0.05
180	10.50835	0.00015	1160.389	0.05
210	10.50976	0.00014	1160.856	0.05
240	10.51112	0.00014	1161.308	0.05
270	10.51287	0.00015	1161.886	0.05
300	10.51457	0.00015	1162.452	0.05
330	10.51644	0.00015	1163.070	0.05
360	10.51800	0.00012	1163.589	0.04
390	10.51994	0.00014	1164.234	0.05
420	10.52173	0.00015	1164.827	0.05
450	10.52334	0.00015	1165.360	0.05
480	10.52503	0.00016	1165.924	0.05
510	10.52715	0.00015	1166.628	0.05
540	10.52891	0.00015	1167.214	0.05
570	10.53075	0.00011	1167.824	0.03
600	10.53302	0.00015	1168.581	0.05
630	10.53535	0.00015	1169.357	0.05
660	10.53742	0.00011	1170.044	0.04
690	10.53945	0.00014	1170.722	0.05
720	10.54191	0.00012	1171.542	0.04
750	10.54396	0.00015	1172.226	0.05
780	10.54635	0.00011	1173.022	0.04
810	10.54979	0.00015	1173.932	0.04
840	10.55171	0.00015	1174.813	0.05
870	10.55441	0.00015	1175.714	0.05
900	10.55663	0.00015	1176.457	0.05
930	10.55990	0.00015	1177.551	0.05
960	10.56255	0.00016	1178.438	0.05
990	10.56570	0.00014	1179.492	0.05
1020	10.56847	0.00014	1180.420	0.05
1050	10.57159	0.00014	1181.465	0.05
1080	10.57455	0.00010	1182.459	0.05
1110	10.57607	0.00010	1182.968	0.05
1140	10.57653	0.00010	1183.122	0.05

1170	10.57403	0.00010	1182.284	0.05
1200	10.57071	0.00010	1181.170	0.05

Table S2. TECs of $Bi_{1.5}Mg_{0.375}Fe_{0.375}Ta_{1.5}O_{7-\Delta}$ at different temperatures

T, ˚C	TECs×10^6, °C^{-1}
30	3.611
90	3.946
150	4.280
210	4.615
270	4.948
330	5.282
390	5.615
450	5.948
510	6.281
570	6.613
630	6.945
690	7.277
750	7.608
810	7.938
870	8.268
930	8.598
990	8.927
1050	9.256
